# Supplementary material for: The Systems Biology Research Tool: evolvable open-source software
Source: BMC Syst Biol. 2008 Jun 29;2:55. doi: 10.1186/1752-0509-2-55 (PMC2446383; doi:10.1186/1752-0509-2-55)
Supplement: Additional file 1 — SBRT Archive. An archive of the current version of the Systems Biology Research Tool. [file 1752-0509-2-55-S1.zip › sbrt-1.4.0/doc/developers_guide/api/index-files/index-15.html]

O-Index


|  |  |  |
| --- | --- | --- |
| **Overview** \| Package \| Class \| Use \| **Tree** \| **Deprecated** \| **Index** \| **Help** | |  |
| **PREV LETTER**   **NEXT LETTER** | **FRAMES**    **NO FRAMES**     **All Classes** |  |


A B C D E F G H I J K L M N O P Q R S T U V W X Y Z 

---


## **O**

**OBJ\_FUNCT\_FILE** - Static variable in class sbrt.shell.mng.fba.ObjectiveFunctionManager: The keyword used to denote the file containing objective functions. **OBJ\_FUNCT\_FILE\_FORMAT** - Static variable in class sbrt.shell.mng.fba.ObjectiveFunctionManager: The keyword used to denote the format of the file containing objective functions. **OBJ\_VALUE** - Static variable in class sbrt.shell.text.fba.FbaOptHeaderFormatterV1: The header used to indicate the value of the objective function. **OBJ\_VALUE** - Static variable in class sbrt.shell.text.fba.FbaOptHeaderParserV1: The header used to indicate the value of the objective function. **objectIterator()** - Method in class sbrt.shell.io.SerializedInputFile: Returns an object iterator over this file. **OBJECTIVE\_FUNCTION** - Static variable in class sbrt.shell.mng.fba.FbaOptManager: The keyword used to denote an objective function. **ObjectiveFunctionAnalysis** - Class in sbrt.kernel.fba.proc: This class is used to perform objective function experiments. **ObjectiveFunctionAnalysis(FbaOptComputer)** - Constructor for class sbrt.kernel.fba.proc.ObjectiveFunctionAnalysis: Constructs a new objective function experiment. **ObjectiveFunctionManager** - Class in sbrt.shell.mng.fba: This class is used to manage objective function experiments. **ObjectiveFunctionManager()** - Constructor for class sbrt.shell.mng.fba.ObjectiveFunctionManager: **OBSTRUCTED\_MOVES\_FILE\_NAME** - Static variable in class sbrt.shell.mng.fba.SvCdHarFluxVectorManager: The keyword used to denote the name of a file to which the number of obstructed moves will be written for each set of constraint variations. **OFF** - Static variable in class sbrt.kernel.fba.IrrevRxnFluxomeV1: The constraint used by default for all source reactions: [0, 0]. **ON** - Static variable in class sbrt.kernel.fba.IrrevRxnFluxomeV1: The constraint used by default for all internal and sink reactions: [0, Infinity). **oppositeSign(double, double)** - Static method in class sbrt.kernel.util.Util: Indicates if the provided numbers are of opposite sign. **oppositeSign(int, int)** - Static method in class sbrt.kernel.util.Util: Indicates if the provided numbers are of opposite sign. **OPTIMIZATION\_SENSE** - Static variable in class sbrt.shell.mng.fba.FbaOptManager: The keyword used to denote the optimization sense, or direction. **OptimizationSense** - Enum in sbrt.kernel.math.optimization: This enum type is used to represent the sense, or direction, of an optimization problem. **optimize()** - Method in class sbrt.ext.cplex.CplexSolver: Computes the optimal value of the current objective function in the direction of the current optimization sense. **optimize()** - Method in class sbrt.ext.glpk.GlpkSolver: Computes the optimal value of the current objective function in the current optimization sense. **optimize()** - Method in interface sbrt.kernel.math.optimization.ProgramSolver: Computes the optimal value of the current objective function in the direction of the current optimization sense. **optionalKeywords** - Static variable in class sbrt.shell.mng.AbstractProcessManager: The set of optional keywords in the `ManagerInput` objects supplied to instances of this class. **optionalKeywords** - Static variable in class sbrt.shell.mng.algebra.LinearCombConverterManager: The set of keywords understood, but not required, by this process manager. **optionalKeywords** - Static variable in class sbrt.shell.mng.algebra.LinearSolverManager: The set of keywords understood, but not required, by this process manager. **optionalKeywords** - Static variable in class sbrt.shell.mng.algebra.MatrixConverterManager: The set of keywords understood, but not required, by this process manager. **optionalKeywords** - Static variable in class sbrt.shell.mng.algebra.MultipleVectorsConverterManager: The set of keywords understood, but not required, by this process manager. **optionalKeywords** - Static variable in class sbrt.shell.mng.algebra.SingleVectorConverterManager: The set of keywords understood, but not required, by this process manager. **optionalKeywords** - Static variable in class sbrt.shell.mng.comb.SingleElementUnionManager: The set of keywords understood, but not required, by this process manager. **optionalKeywords** - Static variable in class sbrt.shell.mng.comb.StrictSingleElementUnionManager: The set of keywords understood, but not required, by this process manager. **optionalKeywords** - Static variable in class sbrt.shell.mng.fba.BiggSbmlReaderManager: The set of keywords understood, but not required, by this process manager. **optionalKeywords** - Static variable in class sbrt.shell.mng.fba.BiggSbmlTranslaterManager: The set of keywords understood, but not required, by this process manager. **optionalKeywords** - Static variable in class sbrt.shell.mng.fba.CatalystDeletionManager: The set of optional keywords in the `ManagerInput` objects supplied to instances of this class. **optionalKeywords** - Static variable in class sbrt.shell.mng.fba.CdHarFluxVectorManager: The set of keywords required to be present in the `ManagerInput` objects supplied to instances of this class. **optionalKeywords** - Static variable in class sbrt.shell.mng.fba.CvCatalystDeletionManager: The set of keywords required to be present in the `ManagerInput` objects supplied to instances of this class. **optionalKeywords** - Static variable in class sbrt.shell.mng.fba.CvFbaOptManager: The set of keywords understood, but not required, by instances of this class. **optionalKeywords** - Static variable in class sbrt.shell.mng.fba.CvMsFluxIntervalManager: The set of keywords understood, but not required, by instances of this class. **optionalKeywords** - Static variable in class sbrt.shell.mng.fba.CvObjectiveFunctionManager: The set of optional keywords in the `ManagerInput` objects supplied to instances of this class. **optionalKeywords** - Static variable in class sbrt.shell.mng.fba.CvRevRxnFluxIntervalManager: The set of keywords understood, but not required, by instances of this class. **optionalKeywords** - Static variable in class sbrt.shell.mng.fba.CvRxnDeletionManager: The set of keywords understood, but not required, by instances of this class. **optionalKeywords** - Static variable in class sbrt.shell.mng.fba.CvSimpleFluxIntervalManager: The set of keywords understood, but not required, by instances of this class. **optionalKeywords** - Static variable in class sbrt.shell.mng.fba.ExtremeCurrentManager: The set of optional keywords in the `ManagerInput` objects supplied to instances of this class. **optionalKeywords** - Static variable in class sbrt.shell.mng.fba.FbaManager: The set of keywords understood, but not required, by instances of this class. **optionalKeywords** - Static variable in class sbrt.shell.mng.fba.FbaOptManager: The set of keywords understood, but not required, by instances of this class. **optionalKeywords** - Static variable in class sbrt.shell.mng.fba.FbaSolverManager: The set of keywords understood, but not required, by instances of this class. **optionalKeywords** - Static variable in class sbrt.shell.mng.fba.FluxActivityManager: The set of optional keywords in the `ManagerInput` objects supplied to instances of this class. **optionalKeywords** - Static variable in class sbrt.shell.mng.fba.FluxCapManager: The set of keywords understood, but not required, by instances of this class. **optionalKeywords** - Static variable in class sbrt.shell.mng.fba.FluxPlasticityManager: The set of keywords understood, but not required, by instances of this class. **optionalKeywords** - Static variable in class sbrt.shell.mng.fba.InitialPointManager: The set of keywords understood, but not required, by instances of this class. **optionalKeywords** - Static variable in class sbrt.shell.mng.fba.MetatoolFileWriterManager: The set of keywords understood, but not required, by this process manager. **optionalKeywords** - Static variable in class sbrt.shell.mng.fba.MmaNotebookManager: The set of keywords understood, but not required, by instances of this class. **optionalKeywords** - Static variable in class sbrt.shell.mng.fba.MsFluxIntervalManager: The set of keywords understood, but not required, by instances of this class. **optionalKeywords** - Static variable in class sbrt.shell.mng.fba.MsFluxomeReducerManager: The set of keywords understood, but not required, by instances of this class. **optionalKeywords** - Static variable in class sbrt.shell.mng.fba.NetworkInfoManager: The set of keywords understood, but not required, by instances of this class. **optionalKeywords** - Static variable in class sbrt.shell.mng.fba.ObjectiveFunctionManager: The set of keywords understood, but not required, by instances of this class. **optionalKeywords** - Static variable in class sbrt.shell.mng.fba.PalssonSbmlReaderManager: The set of keywords understood, but not required, by this process manager. **optionalKeywords** - Static variable in class sbrt.shell.mng.fba.PalssonSbmlTranslaterManager: The set of keywords understood, but not required, by this process manager. **optionalKeywords** - Static variable in class sbrt.shell.mng.fba.RandomConstraintsManager: The set of keywords understood, but not required, by instances of this class. **optionalKeywords** - Static variable in class sbrt.shell.mng.fba.RandomEnvManager: The set of keywords understood, but not required, by instances of this class. **optionalKeywords** - Static variable in class sbrt.shell.mng.fba.RandomObjectiveManager: The set of keywords understood, but not required, by instances of this class. **optionalKeywords** - Static variable in class sbrt.shell.mng.fba.ReverseRxnFluxIntervalManager: The set of keywords understood, but not required, by instances of this class. **optionalKeywords** - Static variable in class sbrt.shell.mng.fba.RxnDeletionManager: The set of optional keywords in the `ManagerInput` objects supplied to instances of this class. **optionalKeywords** - Static variable in class sbrt.shell.mng.fba.RxnRemovalManager: The set of keywords understood, but not required, by instances of this class. **optionalKeywords** - Static variable in class sbrt.shell.mng.fba.SbmlWriterManager: The set of keywords understood, but not required, by this process manager. **optionalKeywords** - Static variable in class sbrt.shell.mng.fba.SimpleFluxIntervalManager: The set of keywords understood, but not required, by instances of this class. **optionalKeywords** - Static variable in class sbrt.shell.mng.fba.SimpleRxnFileTransManager: The set of keywords understood, but not required, by this process manager. **optionalKeywords** - Static variable in class sbrt.shell.mng.fba.SlpCycleIdManager: The set of keywords understood, but not required, by instances of this class. **optionalKeywords** - Static variable in class sbrt.shell.mng.fba.StoichVariationManager: The set of keywords understood, but not required, by instances of this class. **optionalKeywords** - Static variable in class sbrt.shell.mng.fba.SvCdHarFluxVectorManager: The set of keywords required to be present in the `ManagerInput` objects supplied to instances of this class. **optionalKeywords** - Static variable in class sbrt.shell.mng.fba.SvInitialPointManager: The set of optional keywords in the `ManagerInput` objects supplied to instances of this class. **optionalKeywords** - Static variable in class sbrt.shell.mng.fba.WwFluxomeReducerManager: The set of keywords understood, but not required, by instances of this class. **optionalKeywords** - Static variable in class sbrt.shell.mng.geom.CdHarManager: The set of keywords understood, but not required, by this process manager. **optionalKeywords** - Static variable in class sbrt.shell.mng.graph\_theory.CycleIdManager: The set of keywords understood, but not required, by instances of this class. **optionalKeywords** - Static variable in class sbrt.shell.mng.graph\_theory.PathIdManager: The set of keywords understood, but not required, by instances of this class. **optionalKeywords** - Static variable in class sbrt.shell.mng.graph\_theory.UniqueCycleIdManager: The set of keywords understood, but not required, by instances of this class. **optionalKeywords** - Static variable in class sbrt.shell.mng.stat.CorrelationManager: The set of keywords understood, but not required, by this process manager. **optionalKeywords** - Static variable in class sbrt.shell.mng.stat.KendallTauManager: The set of keywords understood, but not required, by this process manager. **optionalKeywords** - Static variable in class sbrt.shell.mng.stat.MannWhitneyUManager: The set of keywords understood, but not required, by this process manager. **optionalKeywords** - Static variable in class sbrt.shell.mng.utilities.IntervalComparisonManager: The set of keywords understood, but not required, by this process manager. **optionalKeywords** - Static variable in class sbrt.shell.mng.utilities.VariableParticipationManager: The set of keywords understood, but not required, by instances of this class. **optionalKeywords** - Static variable in class sbrt.shell.mng.utilities.VectorComparisonManager: The set of keywords understood, but not required, by this process manager. **Or**<E> - Class in sbrt.kernel.util: **Or(Collection<E>)** - Constructor for class sbrt.kernel.util.Or: **OUTPUT\_FILE\_FORMAT** - Static variable in class sbrt.shell.mng.AbstractProcessManager: The keyword used to denote the name of an output file format. **OUTPUT\_FILE\_NAME** - Static variable in class sbrt.shell.mng.AbstractProcessManager: The keyword used to denote the name of the output file. **OUTPUT\_FILE\_NAME\_FILE** - Static variable in class sbrt.shell.mng.AbstractProcessManager: The keyword used to denote the name of a file containing a list of file names for other output files. **OutputFile**<T> - Interface in sbrt.shell.io: This interface is used to represent output files.

---


|  |  |  |
| --- | --- | --- |
| **Overview** \| Package \| Class \| Use \| **Tree** \| **Deprecated** \| **Index** \| **Help** | |  |
| **PREV LETTER**   **NEXT LETTER** | **FRAMES**    **NO FRAMES**     **All Classes** |  |


A B C D E F G H I J K L M N O P Q R S T U V W X Y Z 

---
